# Association between social media use, social appearance anxiety, and eating disorder risk in adolescents

**DOI:** 10.3389/fnut.2026.1812469

**Published:** 2026-10-15

**Authors:** Gülin Öztürk Özkan, Melis Nur Yüce, Beyza Karagöl

**Affiliations:** Faculty of Health Science, Department of Nutrition and Dietetics, İstanbul Medeniyet University, İstanbul, Türkiye

**Keywords:** adolescents, eating disorders, social appearance anxiety, social media use, social media use continuity

## Abstract

**Introduction:**

This study aimed to examine the relationships between social media use and social appearance anxiety, eating behavior, and eating disorders in adolescents.

**Methods:**

In this study, a questionnaire including the social media use scale, social appearance anxiety scale, and eating disorder assessment questionnaire-short form was administered face-to-face to 309 adolescents.

**Results:**

In total, 47.2% of participants were girls and 52.8% were boys, with an average age of 16.0 ± 0.9 years. The most commonly used social media applications are WhatsApp (83.2%) and Instagram (81.2%). The mean social appearance anxiety score of the girls (38.1 ± 16.8) was higher than that of the boys (30.8 ± 13.9). There was a positive relationship between social appearance anxiety and continuity of social media use, as well as the risk of eating disorders (*p* < 0.05). There was also a positive relationship between social media use continuity and the risk of eating disorders (*p* < 0.05). The continuous use of social media increased the likelihood of social appearance anxiety (*β* = 0.963, 95% CI: 0.520–1.406); increased anxiety about social appearance was associated with a higher likelihood of developing an eating disorder (*β* = 0.068, %95 CI: 0.053–0.083) (*p* < 0.05).

**Conclusion:**

Increased social media use in adolescents is associated with higher levels of social appearance anxiety and an increased risk of eating disorders. Educating adolescents about social media use may help reduce the incidence of eating disorders.

## 1 Introduction

Many developmental changes occur during adolescence (1). Adolescence is a period of preparation for adulthood, characterized by social, psychological, and physical changes and identity development (2). During this period, adolescents attempt to adapt to the changes that occur in themselves, while also striving to shape their identity and develop a sense of self. One of the most significant changes that occurs during adolescence is physical development. Changes in appearance can excessively preoccupy adolescents. In addition, they consider other people’s opinions about their own bodies. Currently, there is an increased sensitivity to appearance. The majority of individuals want to be admired and glamorized. Not being liked by others around them causes anxiety in individuals. This anxiety is called social appearance anxiety (1).

Social media is recognized as a tool with numerous benefits, is capable of increasing interaction between individuals, and occupies an important place in daily life (3, 4). For adolescents, communicating with their friends is an important necessity, and social media provides the opportunity to maintain this communication anytime and anywhere (5). For this reason, it has become one of the most important communication channels for adolescents and young people. It has been reported that 95% of adolescents use social media (3, 5). In parallel with the use of social media, an increase in mental health problems among adolescents has also been reported (6). One of the reasons for this is that adolescents constantly compare themselves with their friends (5).

Social appearance anxiety is a type of social anxiety and is defined as the anxiety and stress experienced during the evaluation of one’s appearance by others. Social appearance anxiety arises from the negative evaluation of one’s own appearance and body image (1). Social appearance anxiety can increase the risk of eating disorders by leading to body dissatisfaction (7). During adolescence, the risk of social appearance anxiety may increase (8). It has been reported that the media promotes social appearance anxiety and that the portrayal of perfect men and women can contribute to an increase in anxiety (7).

Adolescence is a period in which dramatic mental, physical, and emotional changes occur, and nutritional habits are also formed. The use of social media may affect eating habits, and adolescents may be influenced toward unhealthy eating. Disturbances in eating behavior may contribute to the development of eating disorders (9). Eating disorders are chronic and treatment-resistant physical and mental illnesses with high mortality rates (10, 11). The etiology is complex and poorly understood, and the condition often follows a chronic course (12). Eating disorders affect all age groups, ethnicities, body sizes, socioeconomic statuses, and religions, regardless of sex (10). Between 1999 and 2022, 22.36% of children and adolescents were reported to have disordered eating (13).

The incidence of eating disorders has increased over the last 40 years, in parallel with the increase in social media use among adolescents (3, 12, 14). The relationship between social media and eating disorders has been frequently studied in recent years (15). It has been shown that social media use can increase concerns about body image. Social media users compare their appearance with that of other individuals and may edit their photos. According to body image theories, one way to transmit social appearance ideals is through the media. Through the media, pressures regarding appearance are placed on individuals, and comparisons are made. As a result, negative thoughts about body image and body dissatisfaction arise. Body dissatisfaction forms a basis for the development of eating disorders. Social media content encourages weight loss, which may lead to eating disorder behaviors and may have negative effects on body image and the achievement of a healthy weight (4).

This study was conducted to examine the relationships between adolescents’ social media use, social appearance anxiety, and the risk of eating disorders. No studies have been found in Türkiye that focus on the continuity of social media use and eating disorders among adolescents. Furthermore, there is only one study that focuses on the continuity of social media use and social appearance anxiety. This study aims to contribute to filling this gap in the literature.

## 2 Materials and methods

### 2.1 Study population

This study was conducted between February and April 2024 with the participation of a total of 309 high school students to examine the relationships between social media use, social appearance anxiety, and the risk of eating disorders among adolescents. The sample size was calculated using the G*Power (v3.1.9.2) (effect size (*f*^2^) = 0.15, *α* = 0.05) program. This effect size (*f*^2^ = 0.15) was determined according to the criteria proposed by Cohen (16) for the social and behavioral sciences. To maximize the sensitivity of the analyses in identifying the complex, multifactorial relationships among social media use, social appearance anxiety, and eating disorder risk among adolescents, a small-to-medium effect size was adopted. It was determined that a minimum of 285 students was required to detect significant relationships in correlation and regression analyses. The study was conducted in two randomly selected high schools in Istanbul. There were a total of 1,457 students in these two schools. A total of 309 of these students volunteered to participate in the study. The adolescents included in the study were physically and mentally healthy. A total of 47.2% (*n* = 146) of the participants were girls, and 52.8% (*n* = 163) were boys, and the mean age was 16.0 ± 0.9 years. The mean body mass index (BMI) percentile-for-age was 47.6 ± 27.0% in girls and 62.9 ± 27.5% in boys. Overall, 78.3% of the participants (*n* = 242) were of a healthy weight, namely 87.0% of the girls (*n* = 127) and 70.6% of the boys (*n* = 115), while 11.2% (*n* = 36) were overweight, namely 8.2% of the girls (*n* = 12), and 14.7% of the boys (*n* = 24) (Table 1) (*χ*^2^ = 15.618, *p* < 0.05).

**Table 1:** General characteristics of the participants.

	Girls (*n* = 146)		Boys (*n* = 163)		Total (*n* = 309)			*p*
	*n*	%	*n*	%	*n*	%		
Participants	146	47.2	163	52.8	309	100.0		
Age (mean, SD)	15.9 (0.9)		16.0 (0.9)		16.0 (0.9)			0.668
Body mass index-for-age percentiles (mean, SD)	47.6 (27.0)		62.9 (27.5)		55.7 (28.3)			**<0.001** ^a^^*^
									***n***	**%**	***n***	**%**	***n***	**%**	***x***_2_	***p***
Grade	9th	48	32.9	47	28.8	95	30.7	0.782	0.676
11th	62	42.5	70	42.9	132	42.7
12th	36	24.7	46	28.2	82	26.5
Mother’s education								
Primary school	32	21.9	40	24.5	72	23.3	1.863	0.601
Secondary school	34	23.3	28	17.2	62	20.1		
High school	55	37.7	64	39.3	119	38.5		
Undergraduate	25	17.1	31	19.0	56	18.1		
Father’s education								
Primary school	22	15.1	27	16.6	49	15.9	2.467	0.481
Secondary school	22	15.1	30	18.4	52	16.8		
High school	71	48.6	65	39.9	136	44.0		
Undergraduate	31	21.2	41	25.2	72	23.3		
Mother’s occupation								
Officer	8	5.5	6	3.7	14	4.5	3.123	0.681
Worker	22	15.1	20	12.3	42	13.6		
Self-employment	11	7.5	11	6.7	22	7.1		
Housewife	85	58.2	104	63.8	189	61.2		
Retired	6	4.1	3	1.8	9	2.9		
Other	14	9.6	19	11.7	33	10.7		
Father’s occupation								
Officer	18	12.3	15	9.2	33	10.7	6.431	0.169
Worker	38	26.0	48	29.4	86	27.8		
Self-employment	44	30.1	33	20.2	77	24.9		
Retired	15	10.3	26	16.0	41	13.3		
Other	31	21.2	41	25.2	72	23.3		
Body mass index-for-age percentiles								
Underweight	4	2.7	5	3.1	9	2.9	15.618	**0.004** ^b^^*^
Healthy weight	127	87.0	115	70.6	242	78.3		
Overweight	12	8.2	24	14.7	36	11.7		
Obesity	3	2.1	17	10.4	20	6.5		
Severe obesity	0	0.0	2	1.2	2	0.6		

a Independent samples *t-*test.

b Chi-square test.

*p < 0.05 indicates statistical significance.

In this cross-sectional study, a face-to-face questionnaire form was used with the participants. This form included 11 questions about general information and 4 questions about social media use. The general information section of the survey form included gender, current grade level, parents’ education status, and occupation information. Questions related to social media usage included daily social media usage time, social media platforms used, use of filters or effects for photos, and whether friends’ opinions were sought when sharing photos. In this study, participants were asked which social media platforms they used. They were asked to list all of the social media platforms they use, without any limit on the number. In addition, the questionnaire included a social media use scale (8 questions), a social appearance anxiety scale (16 questions), and an eating disorder evaluation questionnaire-short form (EDE-Q-13) (13 questions). The participants and their families were informed about the study, and their consent was obtained. The height and body weight of the students were measured, and body mass index percentile-for-age was calculated. Body mass index-for-age percentile was classified as underweight if it was below the 5th percentile, healthy weight if it was between the 5th and 85th percentiles, overweight if it was between the 85th and 95th percentiles, obese if it was at or above the 95th percentile, and severely obese if it was at or above 120% of the 95th percentile (17).

### 2.2 Social media use scale

The social media usage scale was developed by Deniz et al. (18) in a study involving secondary school students (54.2% of whom were girls). The scale consists of the subscales ‘continuity of social media use’ (four items) and ‘social media use competence’ (four items) and contains a total of eight items. The concept of social media continuity addressed in this study encompasses not only the duration of social media use throughout the day but also the habitual nature and continuity of access to social media platforms. Due to this characteristic, it reflects the psychological burden associated with social media use. Sample items for this construct include *‘I always check my social media accounts immediately before sleeping and right after waking up’* and *‘I remain continuously online/active through my mobile devices (tablet, phone, etc.)’*. Furthermore, items assessing social media use competence include statements such as *‘I can manage all my tasks using social media and the internet’* and *‘I can simultaneously use devices like tablets or smartphones while performing other tasks’.* The scale uses a five-point Likert format, with response options ranging from 1 (not at all suitable for me), 2 (slightly suitable for me), 3 (moderately suitable for me), 4 (very suitable for me), to 5 (completely suitable for me). The Cronbach’s alpha internal consistency coefficient of the scale was calculated as 0.824. A high total score on the scale indicates intensive social media use (18).

### 2.3 Social appearance anxiety scale

This scale was developed by Hart et al. (19) to assess the anxiety experienced by individuals due to their physical appearance. The reliability and validity study of the scale in Turkish was conducted by Doğan (20), and the internal consistency coefficient for the reliability of the scale was 0.930. There are 16 items in the scale and, in the scale-based scoring, a five-point Likert scale is used for each question, with responses rated as 1 (not at all appropriate), 2 (not appropriate), 3 (somewhat appropriate), 4 (appropriate), and 5 (completely appropriate). For the first item, the scoring is reversed. The social appearance anxiety scale includes cognitive, emotional, and behavioral statements about individuals’ appearance anxiety. An increase in the total score obtained at the end of the scoring is considered an indicator of a decrease in self-esteem and indicates a high level of anxiety (21).

### 2.4 Eating Disorder Examination Questionnaire-Short Form (EDE-Q-13)

In this study, the Eating Disorder Examination Questionnaire-Short Form (EDE-Q-13), a screening tool for eating disorders, was used. The eating disorder assessment questionnaire was developed by Fairburn and Beglin (22). The Turkish validity and reliability study of the scale was conducted by Esin and Ayyıldız (23). The Cronbach’s alpha coefficient of the scale was calculated as 0.890. The questionnaire, which evaluates eating behaviors over the past 28 days, consists of 13 items across 5 subdimensions: Eating Restraint (ER), Shape and Weight Over-Evaluation (SWO), Body Dissatisfaction (BD), Binging, and Purging. The scale uses a seven-point Likert format ranging from 0 (never), 1 (1–5 days), 2 (6–12 days), 3 (13–15 days), 4 (16–22 days), 5 (23–27 days), to 6 (every day). The scale score is based on the scores obtained from each subscale and the total score obtained from all the items. A higher score indicates the presence of eating-related psychopathology (23).

### 2.5 Ethical approval

The study was conducted in accordance with the Declaration of Helsinki, and approved by the Ethics Committee of Istanbul Medeniyet University Social and Human Sciences (No: 2024/1 and date of approval: 23 January 2024) for studies involving humans. Informed consent was obtained from all subjects involved in the study.

*Consent to Participate declaration:* Informed consent was obtained from the parents of the adolescents before participating in this study. All procedures performed in studies involving human participants were in accordance with the ethical standards of the institutional and/or national research committee and with the 1964 Declaration of Helsinki and its later amendments or comparable ethical standards.

### 2.6 Statistical analysis of the data

Statistical evaluation of the data was performed via the SPSS 27 (Statistical Package for the Social Sciences) program. Descriptive statistical methods such as the mean, standard deviation, frequency, and percentage were also used in the evaluation. The normality of the distribution of the data was tested via the Shapiro–Wilk test and graphical analysis. Qualitative and quantitative data were evaluated via the Chi-square test, independent samples *t*-test, Pearson correlation analysis, multiple linear regression analysis, and univariate and multivariate regression analyses. When designing the regression models, the independent variables included in this study—gender, age, parents’ education level, and social media usage—were taken into account. The effect of these variables on social appearance anxiety was examined. In another regression model, in addition to the same independent variables, the relationship between social media usage continuity, competence, and social appearance anxiety with eating disorders was evaluated. Thus, the study aimed to examine the relationships between the variables. Statistical significance was accepted at a *p*-value of <0.05.

## 3 Results

Table 2 shows the mean and standard deviation values of adolescents’ social media use status and social media use scale, social appearance anxiety, and eating disorder assessment scale scores. Accordingly, 81.2% (*n* = 251) of the adolescents used Instagram, and 83.2% (*n* = 257) used WhatsApp. A total of 31.3% (*n* = 51) of the boys and 37.0% (*n* = 54) of the girls preferred to use Snapchat applications (*p* < 0.05). A total of 17.8% of the girls (*n* = 26) and 8.0% of the boys (*n* = 13) used social media effects (*p* < 005). A total of 72.6% of the girls (*n* = 106) and 38.0% of the boys (*n* = 62) stated that they considered the opinions of friends about the photos important (*p* < 0.05). The mean social media proficiency score was 11.8 ± 3.5 for girls and 12.6 ± 3.8 for boys (*p* < 0.05). The mean score of social appearance anxiety was 38.1 ± 16.8 for girls and 30.8 ± 13.9 for boys (*p* < 0.05).

**Table 2:** Adolescents’ social media use status and mean, and standard deviation values of social media use scale scores, social appearance anxiety scores, Dutch eating behavior questionnaire scores, and eating disorder assessment scale scores.

	Girls (*n* = 146)		Boys (*n* = 163)		Total (*n* = 309)			
	*n*	%	*n*	%	*n*	%			*χ* ^2^	*p*
Daily social media usage time								
0–1 h	13	8.9	19	11.7	32	10.4	2.292	0.682
1–3 h	51	34.9	63	38.7	114	36.9		
3–5 h	59	40.4	55	33.7	114	36.9		
5–7 h	18	12.3	18	11.0	36	11.7		
7–9 h	5	3.4	8	4.9	13	4.2		
Social media app								
Facebook	1	0.7	8	4.9	9	2.9	4.857	**0.027** ^**a**^ *
Instagram	121	82.9	130	79.8	251	81.2	0.492	0.290
X	25	17.1	51	31.3	76	24.6	8.332	**0.003** ^a^*
TikTok	59	40.4	52	31.9	111	35.9	2.432	0.075
Snapchat	54	37.0	21	12.9	75	24.3	24.342	**<0.001** ^a^*
WhatsApp	124	84.9	133	81.6	257	83.2	0.613	0.265
Use of social media effects								
Yes	26	17.8	13	8.0	39	12.6	6.752	**0.007** ^a^*
No	120	82.2	150	92.0	270	87.4		
Caring about friends’ opinions about social media photos								
Yes	106		62	38.0	168	54.4	37.091	**<0.001** ^a^*
No	40		101	62.0	141	45.6		

	Mean	SD	Mean	SD	Mean	SD		*p*
Social media use scale								
Continuity score	11.8	3.9	11	3.6	11.4	3.8		0.052
Proficiency score	11.8	3.5	12.6	3.8	12.2	3.6		**0.045** ^b^*
Total score	23.6	6.5	23.6	6.1	23.6	6.3		0.998
Social appearance anxiety scale score	38.1	16.8	30.8	13.9	34.3	15.7		**<0.001** ^b^*
Eating Disorder Examination Questionnaire-Short Form (EDE-Q-13)								
Eating restraint	1.6	1.6	1.3	1.6	1.4	1.6		0.124
Shape and weight over-evaluation	2.1	1.7	1.9	1.6	1.9	1.7		0.355
Body dissatisfaction	2.3	1.9	1.9	1.6	2.1	1.9		0.097
Binging	0.6	0.8	0.6	0.8	0.6	0.8		0.711
Purging	0.1	0.3	0.2	0.5	0.2	0.4		0.167
Total score	3.1	2.4	2.7	2.1	2.9	2.3		0.212

a Chi-square test.

b Independent samples *t*-test.

*p < 0.05 indicates statistical significance.

Table 3 shows the correlation analysis results between social media use, social appearance anxiety, and risk of eating disorders. Accordingly, there was a positive correlation between social appearance anxiety and social media use continuity (*r* = 0.252), eating restraint (*r* = 0.317), shape and weight over-evaluation (*r* = 0.442), body dissatisfaction (*r* = 0.437), binging (*r* = 0.201), purging (*r* = 0.227), and risk of eating disorders (*r* = 0.458) (*p* < 0.05). There was a positive relationship between social media continuity and shape and weight over-evaluation (*r* = 0.241), body dissatisfaction (*r* = 0.098), and the risk of eating disorders (*r* = 0.224) (*p* < 0.01).

**Table 3:** Correlation analysis.

	Social appearance anxiety		Social media use					
	*r*	*p*	Continuity		Proficiency		Total score	
			*r*	*p*	*r*	*p*	*r*	*p*
Social media use								
Continuity	0.266	<0.001**	1.000		0.812	<0.001**	0.848	<0.001**
Proficiency	0.043	0.456	0.409	<0.001**	1.000		0.043	0.456
Total score	0.199	<0.001**	0.848	<0.001**	0.043	0.456	1.000	
Eating Disorder Examination Questionnaire-Short Form (EDE-Q-13)								
Eating restraint	0.317	<0.001**	0.098	0.086	0.011	0.842	0.075	0.187
Shape and weight over-evaluation	0.442	<0.001**	0.241	<0.001**	0.066	0.247	0.191	0.001**
Body dissatisfaction	0.437	<0.001**	0.201	<0.001**	0.054	0.340	0.163	0.004**
Binging	0.201	<0.001**	0.098	0.086	0.082	0.153	0.102	0.073
Purging	0.227	<0.001**	0.012	0.834	0.038	0.502	0.031	0.587
Total score	0.458	<0.001**	0.224	<0.001**	0.079	0.166	0.190	0.001**

Pearson correlation analysis.

* *p* < 0.05 indicates statistical significance.

**p <0.01 indicates statistical significance.

Table 4 shows the results of the regression analysis on variables that may affect social appearance anxiety. Variables that may affect social appearance anxiety (gender, age, mother’s and father’s education level, and social media usage frequency) were included in the multiple linear regression model using the enter method. The multiple linear regression model was statistically significant, *F(4,304)* **=** 27.22, *p* < 0.001, accounting for 26.4% of the variance in social appearance anxiety (*R*^2^ = 0.264). The independent variables affecting social appearance anxiety are gender, age, and social media usage continuity. It has been determined that social appearance anxiety decreases in boys (*B* = −6.295, 95% CI: −9.624 to −2.966) and with increasing age (*B* = −2.853, 95% CI: −4.687 to −1.020), while anxiety levels increase with increasing social media usage continuity (*B* = 0.963, 95% CI: 0.520–1.406) (*p* < 0.001).

**Table 4:** Univariate and multivariate regression analyses for social appearance anxiety.

Predictor	Univariate				Multivariate			
	*B*	SE	95% CI	*p*	*B*	SE	95% CI	*p*
Gender (Ref: Girls)	−7.276	1.479	−10.717 to −3.835	**<0.001**	−6.295	1.722	−9.624 to −2.966	**<0.001**
Age	0.051	0.977	0.008–0.352	**0.003**	−2.853	0.947	−4.687 to −1.020	**0.002**
Body mass index-for-age percentiles	0.023	0.032	−0.033 to 0.092	0.357				
Mother’s education (Ref: Primary school)	0.005	0.004	0.000–0.973	**0.049**	−0.892	2.268	−2.669 to 0.886	0.324
Father’s education (Ref: Primary school)	0.007	0.011	0.000–3.200	0.111				
Mother’s occupation (Ref: Housewife)	8.799	3.533	0.002–49246.502	0.620				
Father’s occupation (Ref: Officer)	5.326	3.327	0.009–3130.970	0.606				
Social media use scale-continuity	2.872	0.232	1.821–4.530	**<0.001**	0.963	0.256	0.520–1.406	**<0.001**
Social media use scale-proficiency	1.266	0.246	0.780–2.055	0.339				

*p* < 0.05 indicates statistical significance.

Table 5 shows the results of the regression analysis on factors affecting the risk of eating disorders. Variables that could affect the total score of the Eating Disorder Examination Questionnaire-Short Form (EDE-Q-13) (gender, age, parents’ occupations, BMI-for-age percentiles, social media usage continuity and proficiency, and total social appearance anxiety score) were evaluated using the multiple linear regression analysis. The multiple linear regression model was statistically significant, *F(4,304)* **=** 31.89, *p* < 0.001, accounting for 29.6% of the variance in eating disorder risk (*R*^2^ = 0.296). The independent variables affecting eating disorder risk are social appearance anxiety and BMI-for-age percentiles. As social appearance anxiety (*B* = 0.068, %95 CI: 0.053–0.083) and BMI-for-age percentiles (*B* = 0.019, %95 CI: 0.010–0.027) increase, the risk of eating disorders was observed to increase (*p* < 0.001).

**Table 5:** Univariate and multivariate regression analysis for the risk of eating disorders.

Predictor	Univariate				Multivariate			
	*B*	SE	95% CI	*p*	*B*	SE	95% CI	*p*
Gender (Ref: Girls)	−0.323	0.258	−0.831–0.185	0.212	−0.189	0.239	−0.276–0.654	0.425
Age	−0.131	0.143	−0.412–0.149	0.358	0.054	0.123	−0.197–0.306	0.671
Body mass index-for-age percentiles	0.017	0.004	0.010–0.025	**<0.001**	0.019	0.004	0.010–0.027	**<0.001**
Mother’s education (Ref: Primary school)	−0.261	0.392	−1.032–0.511	0.507				
Father’s education (Ref: Primary school)	−0.338	0.447	−1.217–0.541	0.450				
Mother’s occupation (Ref: Housewife)	0.790	0.627	−0.444–2.025	0.209				
Father’s occupation (Ref: Officer)	−0.830	0.463	−1.742–0.081	0.074				
Social media use scale-continuity	0.134	0.034	0.068–0.200	**<0.001**	0.054	0.034	−0.015–0.123	0.125
Social media use scale-proficiency	0.066	0.035	−0.004–0.135	0.063	0.012	0.024	−0.047–0.092	0.517
Social appearance anxiety scale score	0.070	0.007	0.056–0.084	**<0.001**	0.068	0.008	0.053–0.083	**<0.001**

*p* < 0.05 indicates statistical significance.

## 4 Discussion

### 4.1 Social media use

In this study, which was conducted to evaluate the relationships among social media use, social appearance anxiety, and the risk of eating disorders in adolescents, the daily social media usage time of adolescents was examined. In one study, 48.9% of young people used social media for 1–3 h, and 32.3% used social media for 4–6 h daily (24). It has been reported that 85% of adolescents aged 12–17 years in Germany spend approximately 3 h a day on social media (25). In a study conducted by Caner et al. (5), 25.7% of adolescents were online for 4 h/day, and 25.1% were online for 3 h/day. The findings of the present study indicate that a substantial majority of adolescents (73.8%) engage with social media for 1–5 h daily, a pattern that aligns with previously reported global trends. This observation reinforces the prevailing view that digital platforms have transitioned into a primary environment for adolescent socialization and interpersonal interaction. The fact that adolescents spend so much time on social media leads to various risks, such as cyberbullying, educational problems, psychological deterioration, and sexual issues (26). An increased use of social media negatively affects mental health (27). Possible consequences of social media use include an increased risk of sleep problems, anxiety, and depression (28, 29).

Social media preferences during adolescence may vary according to gender. The prevalence of social media platforms such as Instagram, Snapchat, TikTok, and Facebook, where visuality is at the forefront, is significant among girls (6). Studies conducted by Sagrera et al. (30) and Wilksch et al. (15) have shown that Instagram usage rates among adolescents range from 48 to 68%. When examining participants’ social media preferences, WhatsApp (83.2%) was the most widely used application, followed by Instagram (81.2%). Although it was observed that girls (82.9%) used this platform at a higher rate than boys (79.8%), the difference was found to be statistically insignificant (*χ*^2^ = 0.492, *p* > 0.05). These findings indicate that messaging and visual-sharing applications play a significant role in adolescents’ communication and social interactions, regardless of gender. The high rate of Instagram usage in this study demonstrates the popularity of visually oriented social media among the young population and is consistent with a previous study (5). Our findings primarily reflect the frequency of social media use. However, there are also studies examining the effects of interactions on social media platforms. For instance, it has been noted that an increase in the frequency of social media use—including use of visually oriented platforms—may be associated with mental health issues such as depression, anxiety, and stress (31). In this recent study, the widespread prevalence of social media use among adolescents underscores the need for a comprehensive investigation into the relationship between digital communication and interaction platforms and health outcomes. When planning initiatives to protect and promote adolescent mental health, social media use—including messaging and visually-oriented applications—must be taken into account.

#### 4.1.1 Social media use and eating disorders

Social media use encourages girls to focus on their physical appearance and contributes to an increased risk of eating disorders (6). There is a positive relationship between the amount of time spent on social media, body dissatisfaction, and the risk of eating disorders (15, 32, 33). The relationship between social media and body image anxiety is mediated by social comparison (32). Body dissatisfaction promotes the development of eating disorders (34). In this study, a positive correlation was found between social media usage continuity and body dissatisfaction (*r* = 0.201; *p* < 0.001) and between social media usage continuity and risk of eating disorders (*r* = 0.224; *p* < 0.001) (Table 3). Although the mechanism underlying the observed positive association was not examined in this study, the results of previous studies suggest that social media use increases the risk of eating disorders by contributing to body dissatisfaction and a distorted body image. Although bivariate analyses indicated a significant association between continuous social media use and eating disorder risk, multivariate regression models revealed that this relationship was not direct (Table 5) (*p* > 0.05). These findings suggest that the observed risk is primarily driven by underlying psychological constructs, such as social appearance anxiety and body image. When developing strategies to reduce the risk of eating disorders in adolescents, priority should be given to interventions that address body image and promote body satisfaction, in addition to limiting social media use.

### 4.2 Social appearance anxiety

It has been found that social appearance anxiety is frequently observed in overweight individuals and that there is a direct relationship between BMI and social appearance anxiety. It has been reported that this relationship is particularly noticeable in women (35). In this study, it was found that the prevalence of overweight was higher among boys (14.7%) than among girls (8.2%). In addition, the mean BMI-for-age percentile for boys (62.9 ± 27.5) was found to be significantly higher than that for girls (46.7 ± 27.0) (Table 1) (*p* < 0.05). Although the social appearance anxiety score was significantly higher in girls (38.1 ± 16.8) than in boys (30.8 ± 13.9) (*p* < 0.05), there was no significant difference between girls and boys in terms of body dissatisfaction scores (*p* > 0.05) (Table 2). In this study, the overweight group consisted primarily of male individuals. This finding suggests that the relationship between BMI and social appearance anxiety may differ by gender. According to the data obtained, BMI may be positively associated with social appearance anxiety and body dissatisfaction in girls, but this may not be the case for boys. Based on this finding, it also indicates that BMI is not the only factor affecting the emergence of social appearance anxiety and body dissatisfaction.

During adolescence, physical appearance is accepted as a determinant of acceptance among friends. For girls, this situation seems to be more important. Friends and colleagues play a major role in determining ideal appearance standards (32). Our findings also support this dynamic, as a significant portion of the participants (54.4%) reported prioritizing their peers’ opinions regarding the photos they share. Compared with boys, girls care more about the opinions and thoughts of their friends about their appearance (32). In the present study, while 72.6% of adolescent girls indicated that they cared about peer feedback regarding their visual content, this rate was 38.0% among boys. The fact that girls are sensitive about their appearance aligns with previous studies. These findings suggest that girls use social media not only for communication but also to seek acceptance regarding their appearance. Future studies should examine the impact of social media on the development of eating disorder behaviors, specifically focusing on the role of peer-induced body image anxiety.

#### 4.2.1 Social appearance anxiety and social media use

Social media creates an environment where people’s appearances are compared. This can cause social media users’ body perceptions to be distorted and social appearance anxiety to emerge (32). Social media use can be a significant catalyst for social appearance anxiety. Social appearance anxiety can be greater for women, even if they have similar concerns about their physical attractiveness (36). It has been determined that social appearance anxiety is related to gender (37). In the present study, it was found that the mean social appearance anxiety score of girls (38.1 ± 16.8) was significantly higher than that of boys (30.8 ± 13.9) (Table 2). The findings of studies conducted by Boursier et al. (36) and Çetinkaya et al. (1) also align with this study. These findings indicate that the risk of social appearance anxiety is higher among girls. An increase in social appearance anxiety affects body image (38). This study did not investigate the effect of social appearance anxiety on body image. However, research in this area could help to better understand the relationship between social appearance anxiety, body image, and eating disorders.

A study conducted by Caner et al. (5) revealed that there is a positive relationship between the amount of time spent on social media and social appearance anxiety. In this study, it was determined that there was a positive correlation between social media continuity and social appearance anxiety scores (Table 3) and that continuity in social media use is associated with the occurrence of social appearance anxiety (Table 4). Based on the findings, the continuity of social media use may be associated with an increased risk of social appearance anxiety. Social media usage can negatively affect body image, triggering negative thoughts and anxiety related to appearance (2, 39). Continuity of social media use could increase the likelihood of the occurrence of social appearance anxiety (Table 4) (*p* < 0.05). The results indicate a relationship between the frequency of social media use and higher levels of social appearance anxiety and body dissatisfaction. The relationship between social media usage and body image anxiety may be bidirectional. In adolescents heavily exposed to visual content, the behavior of comparing their own bodies to idealized ones may lead to the development of body image anxiety. On the other hand, adolescents with high body image anxiety may tend to use social media as a tool to seek approval and attention from others regarding their appearance. The cross-sectional nature of the study prevents the establishment of causality. However, the findings suggest that the continuity of social media use and concerns about social appearance are mutually reinforcing factors and may trigger the risk of eating disorders through this process.

#### 4.2.2 Social appearance anxiety and eating disorder

Social appearance anxiety may lead to an increased risk of eating disorders (40). The results of the correlation analysis (Table 3) and regression analysis (Table 5) conducted in this study also indicate that social appearance anxiety is associated with the risk of eating disorders. Social appearance anxiety is a factor that negatively affects mental health (41). Eating disorder behaviors are associated with pathologies related to the psychological state (42). Although current findings suggest that social appearance anxiety is associated with the risk of eating disorders, the underlying mechanisms of this relationship have not been clearly examined in this study. However, the study found that social media use was positively associated with social appearance anxiety, body dissatisfaction, and the risk of eating disorders. The findings of this study indicate that social appearance anxiety is associated with the risk of eating disorders. Anxiety regarding physical appearance, including body image, may increase adolescents’ risk of developing eating disorders. Future comprehensive studies should be designed to shed light on these complex dynamics. In this study, another factor associated with an increased risk of eating disorders in adolescents is BMI-for-age percentile. An increase in BMI-for-age percentile has been found to be associated with an increased risk of eating disorders. In a study conducted by Flament et al. (43), body weight was associated with an increased risk of eating disorders in adolescents. The results of this study indicate that weight gain in adolescents is associated with an increased risk of eating disorders. To reduce the risk of eating disorders in this age group, it is important to plan healthy eating education programs, increase physical activity, and develop policies aimed at weight control in order to improve public health.

## 5 Conclusion

This study examined the associations between social media and adolescents’ risk of eating disorders. Adolescents spend a considerable amount of time on social media. Continuity of social media use is associated with social appearance anxiety and the risk of eating disorders. In this study, the mediating effect of social appearance anxiety on the relationship between social media use and the risk of eating disorders was not tested. This study identified some of the factors linked to social appearance anxiety and eating disorder risk in the regression models used. More extensive studies are needed to identify other factors associated with social appearance anxiety and the risk of eating disorders. Designing social media literacy education programs for individuals in this age group and organizing activities in schools to raise awareness on this subject will lay the groundwork for the development of conscious social media users. Suitable environments can be created and activities organized for adolescents to communicate face-to-face with their friends. In addition, trips, games, and activities that bring family members together can help reduce the amount of time adolescents spend on social media. It is also important for national governments to develop informative policies aimed at raising awareness about conscious social media use. Educating adolescents about social media use and monitoring their usage may be associated with a potential reduction in the risk of eating disorders and related health problems in the long term.

## 6 Limitations

The fact that the study was conducted in only one city and two high schools makes it difficult to generalize the findings. Conducting this study in different cities and high schools would increase the generalizability of the results. A limitation of the present study is that potential confounding factors that may influence both social media use and eating disorder risk, such as mental health status (e.g., depression or anxiety disorders), self-esteem, and socioeconomic status, were not examined. The omission of these variables may have led to an overestimation of the observed relationships or, conversely, a failure to identify existing associations. Incorporating these factors into future research addressing the link between social media use and eating disorders will allow for more robust and reliable interpretations of the findings. The explanatory power of the factors included in the regression models within this study remained relatively limited (13.7 and 25.6%). These values indicate that not all variables influencing the outcomes were accounted for, representing a notable methodological limitation. This finding underscores the need for incorporating a broader range of predictors in future research to enhance the model’s predictive capacity. This study utilized age data from adolescents. In this age group, development does not progress at the same rate for all individuals, and individual differences exist. Physical changes can influence social appearance anxiety. The fact that the participants’ pubertal development stages were not objectively determined may have limited the analysis of the effect of physical characteristics on social appearance anxiety.

In this study, we focused on body image concerns and the risk of eating disorders; however, body dissatisfaction was not directly assessed. Including pubertal stages and body dissatisfaction in future studies on this topic could contribute to a clearer explanation of the relationship between social media use, body image concerns, and the risk of eating disorders.

## Data Availability

The raw data supporting the conclusions of this article will be made available by the authors, without undue reservation.
